# Validation of the full-age spectrum equation in the approximation of glomerular filtration rate in Chinese patients with chronic kidney disease

**DOI:** 10.1080/0886022X.2019.1620773

**Published:** 2019-06-04

**Authors:** Peng Xie, Huan-Li Li, Jian-Min Huang, Ling-Ge Wei

**Affiliations:** a Department of Nuclear Medicine, The Third Hospital, Hebei Medical University, Hebei, People’s Republic of China;; b Department of Ophthalmology, The Hebei General Hospital, Hebei, People’s Republic of China

**Keywords:** Chronic kidney disease, FAS equation, glomerular filtration rate, renal dynamic imaging

## Abstract

**Introduction:** To investigate the validity of the full age spectrum (FAS) equation in determining the glomerular filtration rate (GFR) in Chinese patients with chronic kidney disease (CKD) and to compare the performance of FAS equation and the technetium-99m-diethylene triamine pentaacetic acid (Tc-99m-DTPA) renal dynamic imaging method.

**Methods:** The GFR was determined by three methods in the same day: (a) Tc-99m-DTPA dual plasma sample clearance method (mGFR); (b) FAS equation (eGFR1); (c) Tc-99m-DTPA renal dynamic imaging method (eGFR2). The mGFR was used as the reference standard. The Bland–Altman method, concordance correlation coefficient and regression equation were applied to evaluate the validity of the estimated glomerular filtration rate (eGFR). The bias, precision and accuracy were analyzed to compare the performances of eGFR1 and eGFR2.

**Results:** A total of 162 subjects were enrolled in this study. The eGFR1 was correlated well with mGFR (concordance correlation coefficient was 0.896, *p* < 0.0001) and the regression equation was mGFR = –0.374 + 1.029eGFR1 (*p* < 0001). The Bland–Altman analysis proved good agreement between the eGFR1 and mGFR. In comparison with eGFR2, the eGFR1 showed better performance on bias (–1.22 vs. 8.92, *p* < 0001), precision (15.69 vs. 18.36, *p*  =  0.047) and 30% accuracy (75.31% vs. 59.26%, *p*  =  0.0009) in all participants.

**Conclusions:** The FAS equation is valid in determining the glomerular filtration rate in Chinese patients with chronic kidney disease. The Tc-99m-DTPA renal dynamic imaging method is less accurate than the FAS equation and cannot be employed as the reference method in assessing the performance of FAS equation.

## Introduction

Chronic kidney disease (CKD) is a worldwide problem, and the overall prevalence of CKD in China is 10.8% [1]. It severely impairs patients’ quality of life and leads to significant CKD-related mortality rate [2–4]. Glomerular filtration rate (GFR) is the most important and accurate parameter of kidney function in the course of CKD [5–6]. Accurate measure of GFR quantitates the degree of renal dysfunction. Inulin is not protein-bound, not metabolizable, freely filtered, not reabsorbed, not secreted, so the inulin clearance rate is the gold standard for determining GFR. However, this method cannot be applied routinely because of the inconvenience. Several available methods have been developed to estimate GFR in a simpler manner and at low costs [7–16].

The Chronic Kidney Disease Epidemiology Collaboration (CKD-EPI) equation is widely used to evaluate the GFR with higher accuracy and simpler operating procedure [17–21]. However, this equation is not accurate for the full age spectrum. In 2016, the new equation, full age spectrum (FAS) equation, based on the serum creatinine, was developed and can be applicable to all ages [10]. Chai assessed the performance of FAS equation in the subjects with CKD and indicated that the FAS equation was more accurate and less bias and improved precision than others equations [22]. However, in that study the reference method to determine GFR was ^99m^Tc-diethylene triamine pentaacetic acid (Tc-99m-DTPA) renal dynamic imaging method which was not accurate and could be affected by various factors [23–26]. In this study, we described the implementation of a paired cohort study to validate the applicability of the new FAS equation in determining GFR in Chinese patients with CKD and compare its performance with the Tc-99m-DTPA renal dynamic imaging method and the Tc-99m-DTPA dual plasma sample clearance method [27]. We hypothesize that the FAS equation could be applied to approximate the GFR in Chinese patients with CKD.

## Methods

### Ethics statement

The study protocol was approved by Hebei Medical University ethical committee (No. 2017-027-1), and the written informed consent was obtained from each participant.

### Patients

All the enrolled participants met the diagnostic standard for CKD [28]. The inclusion and exclusion criteria were described elsewhere [23].

#### The GFR measurement by dual plasma sample clearance method (mGFR)

The GFR (mGFR) determined by Tc-99m-DTPA dual plasma sample clearance method was described previously [23], which was calculated from a single exponential formula derived from the blood samples between 2 and 4 h after injection. The mGFR was used as the reference method.

#### GFR measurement by FAS equation (eGFR1)

The serum creatinine (Scr) was automatically measured by the enzymatic IDMS-traceable method using a biochemical analyzer (VITROS 5.1, Johnson Company, USA; reagents from the same company).

The FAS equation was as follows [10]:

For 2 ≦ age ≦ 40 years

eGFR1 = 107.3/(Scr/Q);

For age >40 years

$$\text{eGFR1}=\text{1}0\text{7}.\text{3}/(\text{Scr}/Q)\times {\left(0.\text{988}\right)}^{\left(\text{age}-\text{4}0\right)}$$

*Q* values are the mean or median SCr value for age-/sex-specific healthy populations, listed in the publication [10]. The units of Scr and age are mg/dL and year, respectively.

#### GFR measurement by the renal dynamic imaging method (eGFR2)

GFR determination by the Tc-99m-DTPA renal dynamic imaging method (eGFR2) was described in our previous study [23], which was estimated automatically with the program of the Single Photon Emission Computed Tomography after sketching the region of interesting. The radiotracer and the Single Photon Emission Computed Tomography used were both the same as those in that study.

#### The normalization of mGFR and eGFR2

The mGFR and eGFR2 were normalized for a body surface area of 1.73 m^2^ according to Haycock’s equation [29].

### Statistical analysis

All the statistical analysis was performed with SPSS, version 19.0 (SPSS Inc., Chicago, IL, USA) and Medcale (version 15.2.2 Medcale software, Mariekerke, Belgium). Significance level was set at *p* < 05.

Based on the skewed distribution by the scatter plot, the relationship between eGFR1 and mGFR was assessed with the Spearman correlation and linear regression method. The Bland–Altman method was applied to evaluate the degree of agreement between eGFR1 and mGFR.

The parameters of bias, precision, and accuracy were employed to compare the performance of the eGFR1 and eGFR2. Bias was defined as eGFR minus mGFR. Precision was defined as the standard deviation of the bias. Accuracy was defined as P30, the percentage of GFR (eGFR) values within 30% deviation of the mGFR. Paired *T* test, *F* test and McNemar’s test were used to compare the bias, precision and accuracy respectively.

## Results

### The characteristics of the participants

The demographic and biochemical characteristics of all the 162 participants were shown in Table 1.

**Table 1.** The characteristics of all the patients.

Characteristic (*n* = 162)	Value
Female/male	84/78
Age (years)	56.04 ± 15.39
Weight (kg)	68.23 ± 12.16
Height (cm)	167.73 ± 7.42
BSA (m^2^)	1.73 ± 0.16
BMI（kg/m^2^）	23.67 ± 17.23
Serum creatinine (μmol/L)	180.77 ± 213.76 (ranging from 47 to 1482.8)
Serum urea nitrogen (mmol/L)	9.89 ± 7.43
Volume of 24 h urine (mL)	2401.52 ± 892.67
Causes of CKD [*n*(%)]	
Chronic glomerulonephritis	52 (32.10)
Diabetic nephropathy	36(22.22)
Chronic pyelonephritis	30 (18.51)
Hypertensive nephropathy	21 (12.96)
Urinary tract infection	7 (4.32)
Chronic interstitial nephritis	7 (4.42)
Others	9 (5.56)
CKD stages [*n* (%)]	
1	33 (20.37%)
2	36 (22.22%)
3	44 (27.16%)
4	30 (18.52%)
5	19 (11.73%)
mGFR(mL min^–1^ 1.73 m^–2^)	55.81 ± 35.31 (ranging from 3.54 to 176.29)

Abbreviation: CKD: chronic kidney disease.

There were 84 females and 78 males with a mean age of 56.04 ± 15.39 years in this study. The main etiologies of CKD were chronic glomerulonephritis (52 cases, 32.1%), diabetic kidney disease (36 cases, 22.22%), chronic pyelonephritis (30 cases, 18.51%) and hypertensive nephropathy (21 cases, 12.96%). The average mGFR was 55.81 ± 35.31 (ranging from 3.54 to 176.29) mL min^−1^ 1.73 m^−2^. According to the mGFR values, the patients were divided into five CKD stages in accordance with the K/DOQI guidelines: 33 cases (20.37%) in stage 1, 36 cases (22.22%) in stage 2, 44 cases (27.16%) in stage 3, 30 cases (18.52%) in stage 4, and 19 cases (11.73%) in stage 5.

### The validation of FSA equation

**Figure 1.**
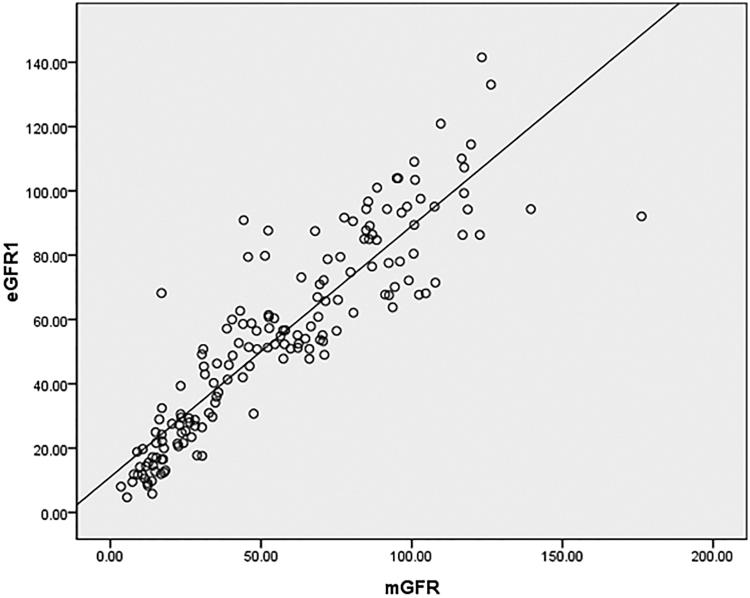
Scatter plots of eGFR1 versus mGFR. Abbreviations: mGFR: the GFR measured by the 99mTc-diethylene triamine pentaacetic acid dual plasma sample clearance method; eGFR1: the GFR estimated by the FAS equation.

**Figure 2.**
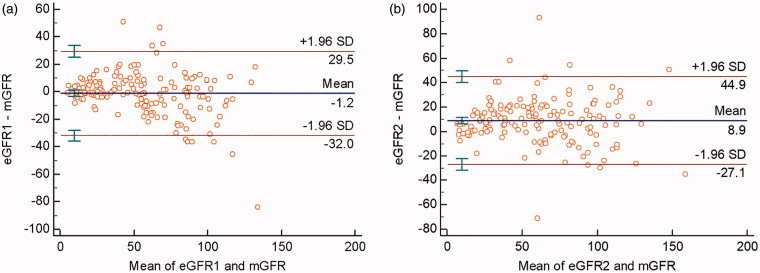
(A) Bland–Altman plot showing the disagreement between eGFR1 and mGFR. The blue line indicates the mean of difference and the red lines represent the 95% limits of agreement. (B) Bland–Altman plot showing the disagreement between eGFR2 and mGFR. The blue line indicates the mean of difference and the red lines represent the 95% limits of agreement. Abbreviations: mGFR: the GFR measured by the 99mTc-diethylene triamine pentaacetic acid dual plasma sample clearance method; eGFR1: the GFR estimated by the FAS equation; eGFR2: the GFR estimated by the Tc-99m-DTPA renal dynamic imaging method.

The average eGFR1 was 54.59 ± 30.74 (ranging from 4.69 to 141.54) mL min^−1^ 1.73 m^−2^. There was no significant difference between eGFR1 and mGFR (*t* = –0.987, *p*  =  0.325). The FAS equation was correlated well with mGFR, and the correlation coefficient of eGFR1 was 0.8962. The scatter plot showed linear correlation relationship between eGFR1 and mGFR (Figure 1). The linear regression was therefore constructed and the regression equation was mGFR = –0.374 + 1.029eGFR1 (*p* < 0.0001). The Bland–Altman plots of eGFR1 against mGFR were shown in Figure 2A. The 95% limit of agreement for the formulas was –32 to 29.5 mL min^−1^ 1.73 m^−2^. The characteristics of the 10 patients that lie outside of the 95% confidence intervals were listed in Table 2. The patients with obesity (higher BMI) were more likely to lie outside of the 95% confidence intervals. The BMI of the 10 patients was higer than of the other 152 patients (26.72 ± 6.73 kg/m^2^ vs. 23.67 ± 17.23 kg/m^2^, *t*  =  2.024, *p*  =  0.045).

**Table 2.** The characteristics of the 10 patients that lie outside of the 95% confidence intervals.

	Gender	Age (years)	Height (cm)	Weight (Kg)	BSA (m^2^)	BMI (kg/m^2^）	Serum creatinine (μmol/L)	Causes of CKD	mGFR	eGFR1
1	Female	55	1.62	65	1.69	24.77	82	Occult nephritis	102.43	67.69
2	Female	64	1.63	104	2.07	39.14	73	Chronic glomerulonephritis	16.98	68.21
3	Female	69	1.60	41	1.37	16.01	59	Urinary tract Infections	45.71	79.45
4	Male	42	1.77	86	2.03	27.45	91	Chronic glomerulonephritis	176.29	92.08
5	Male	47	1.73	66.5	1.79	22.22	90	Chronic interstitial nephritis	52.39	87.65
6	Male	52	1.68	77	1.86	27.28	109	Hypertensive nephropathy	104.67	68.13
7	Male	54	1.68	89	1.98	31.53	84	Hypertensive nephropathy	116.90	86.30
8	Male	56	1.73	62.5	1.74	20.88	99	Occult nephritis	107.85	71.48
9	Male	58	1.75	93	2.08	30.37	80	Chronic glomerulonephritis	122.59	86.34
10	Male	66	1.72	82	1.95	27.72	69	Chronic glomerulonephritis	44.26	90.89

### The comparison between eGFR1 and eGFR2

The performance of the FAS equation compared with Tc-99m-DTPA renal dynamic imaging method is summarized in Table 3. The bias of eGFR1 and eGFR2 was –1.22 and 8.92 mL min^−1^ 1.73 m^−2^, respectively, and the difference was significant (*t* = 6.352, *p* < 0001). The precision of eGFR1 was 15.69 mL min^−1^ 1.73 m^−2^, which was superior to 18.36 mL min^−1^ 1.73 m^−2^ of eGFR2 (*F* = 1.3693, *p* = 0.047). The percentage of P30 of the FAS equation was significantly higher than that of Tc-99m-DTPA renal dynamic imaging method (75.31 vs. 59.26%, *p* = 0.0009). Bland–Altman demonstrated that eGFR1 agreed with mGFR more than eGFR2 agreed with mGFR: 95% confidence intervals for eGFR1–mGFR (61.5) is “tighter” than the 95% confidence intervals for eGFR2–mGFR (72.0) (Figure 2).

**Table 3.** The comparison between eGFR1 and eGFR2.

	eGFR1	eGFR2
Bias (mL min^–1^ 1.73 m^–2^)	–1.22*	8.92
Precision (mL min^–1^ 1.73 m^–2^)	15.69*	18.36
Percentage of P30 (%)	75.31*	59.26

Abbreviations: eGFR1: the GFR estimated by the FAS equation; eGFR2: the GFR estimated by the renal dynamic method.

*p < 0.05 (compared with the value of eGFR2).

## Discussion

Several methods have been developed to estimate the glomerular filtration rate because it cannot be directly determined. In 2016 a new equation based on the level of SCr was developed, named FAS equation, which was applicable to the full age-spectrum [10]. In the study, 14 different datasets, covering 735 children adolescents, 4371 adults and 1764 older adults, were obtained to validate the FAS equation. The validation study revealed that the FAS equation improved validity and continuity across the full age-spectrum and that it could be a reliable alternative [10]. However, it requires more studies to verify whether the new equation is suitable in Chinese patients with CKD because all the datasets were from Europe and the USA. In the present study, the GFR calculated by FAS equation was correlated well to the GFR determined by the dual plasma sample clearance method. We showed that the FAS equation was suitable to determine the GFR in Chinese patients with CKD.

Tc-99m DTPA renal dynamic scintigraphy is widely used to evaluate the renal function because of its simplicity and accuracy. In practice, when impaired renal function is suspected due to various reasons, particularly for the patients with asymmetrical renal dysfunction, renal dynamic imaging is required to assess the split renal function and estimate the single-kidney glomerular filtration rate (GFR). However, the GFR calculated with the Tc-99m-DTPA renal dynamic imaging method might be inaccurate because of the depth and position of the kidney, the selection of region of interest of the background, the presence of tumor of the kidney, the dosage of radiopharmaceutical and the motion artifacts [30–32]. Therefore, the Tc-99m-DTPA renal dynamic imaging method is not suitable to be employed as the reference standard because of its unsatisfactory performance [23,33]. Chai et al. assessed the performance of FAS equation in Chinese subjects with CKD [22]. However, in the study the reference method was the Tc-99m-DTPA renal dynamic imaging method, which might compromise the reliability of the conclusions. In the present study, the Tc-99m-DTPA dual plasma sample clearance method was employed as the reference method, which was more accurate and had been used as the gold standard in several studies [17,23,33,34]. In our study, by comparing the FAS equation with the Tc-99m-DTPA renal dynamic imaging method, we showed that the latter one had worse performance than the former one. The present study revealed that the renal dynamic imaging method could not be employed as the reference method in assessing the performance of FAS equation.

Our future studies will be focused on the following aspects to bring the applicability of our findings to an even higher level. First, we will increase the sample size and take into account different disease stages and patients’ age span in our data analysis. Furthermore, in addition to CKD patients, our future work will also target the validation of the FAS equation in general Chinese population.

In conclusion, the FAS equation is an effective method to determine the GFR of Chinese patients with CKD. The Tc-99m-DTPA renal dynamic imaging method is less accurate than the FAS equation and cannot be employed as the reference method in assessing the performance of FAS equation.
